# Supplementary material for: The B-type cyclin Clb4 prevents meiosis I sister centromere separation in budding yeast
Source: G3 (Bethesda). 2025 May 30;15(9):jkaf121. doi: 10.1093/g3journal/jkaf121 (PMC12405888; doi:10.1093/g3journal/jkaf121)
Supplement: jkaf121_Supplementary_Data [file jkaf121_supplementary_data.zip › Supplemental_Figures_G3-2024-405620.pdf]

Supplementary figures

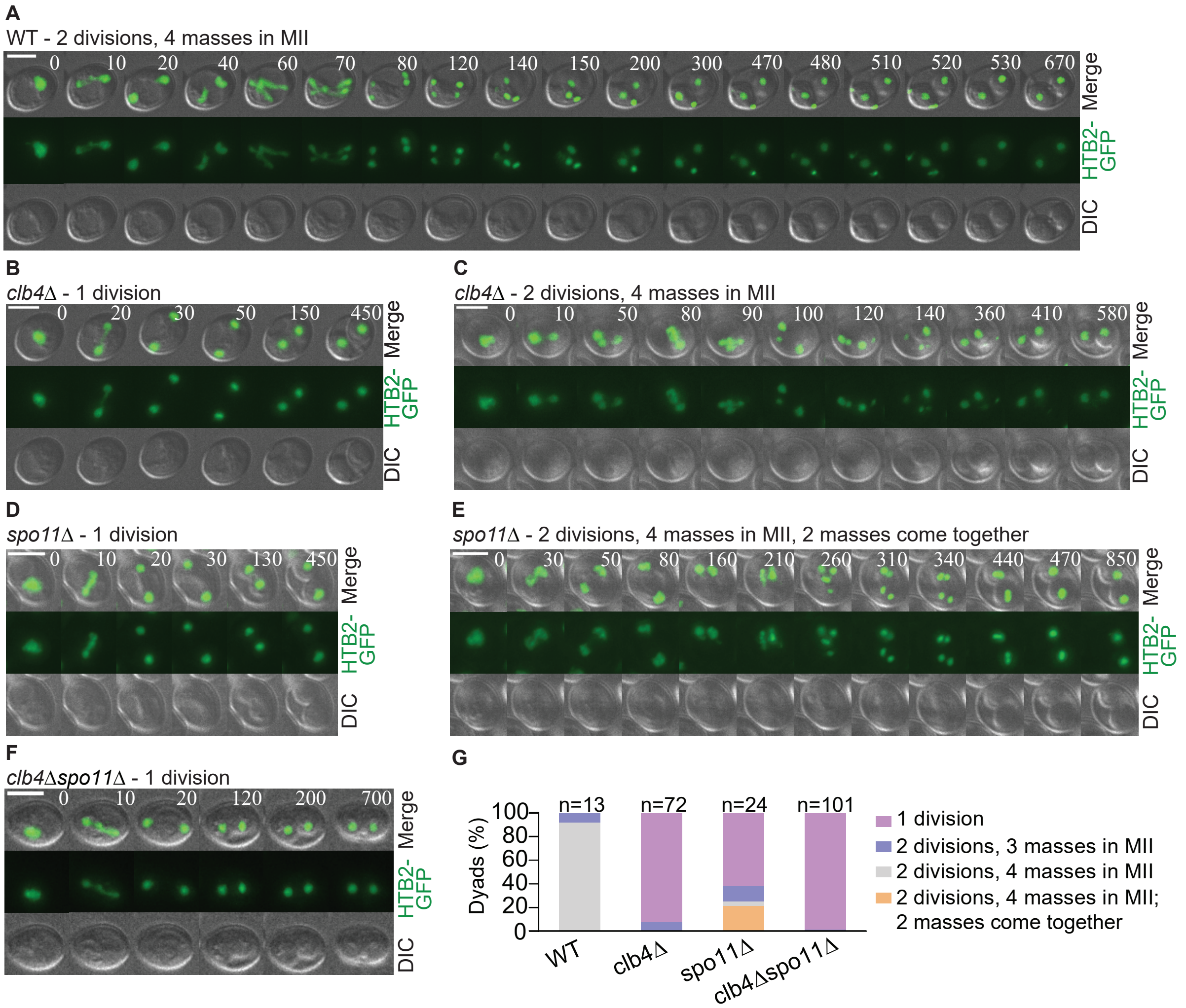

Figure S1. Meiotic chromatin mass divisions in *CLB4* and *SPO11* mutants. (A-F) Representative images from time lapse movies of sporulation with fluorescently labeled histone H2B (HTB2-GFP) in wild-type (WT, LY9981), *clb4*Δ (LY9982), *spo11*Δ (LY10028) and *clb4*Δ *spo11*Δ (LY9983). All deletions were homozygous. **(G)** Fraction of dyads observed forming in time lapse movies that underwent the listed division pattern. The number of dyads formed after 1 or 2 chromatin mass divisions was compared between the following strains using a chi-square test: wild-type (WT) vs. *clb4*Δ, $\chi^2(df = 1, N = 85) = 57.13, p < 0.00001$; *spo11*Δ vs *clb4*Δ *spo11*Δ, $\chi^2(df = 1, N = 125) = 35.12, p < 0.00001$.

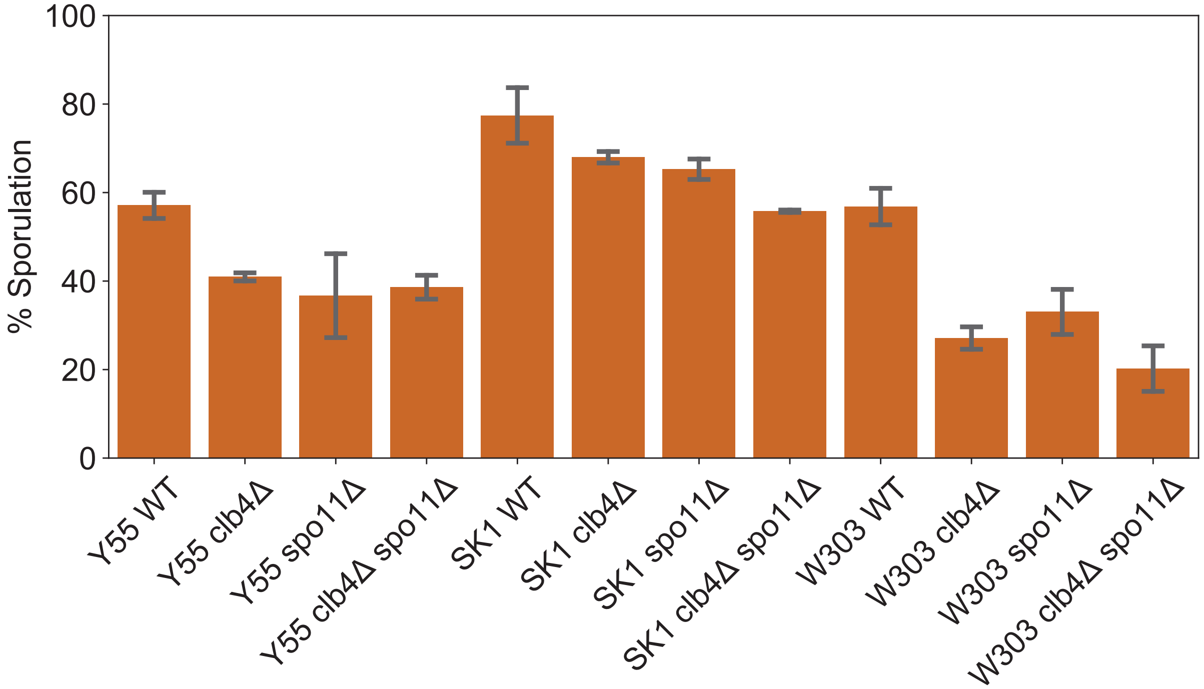

Figure S2. Sporulation fraction of *CLB4* mutants. The fraction of sporulated cells (asci) in the cultures assayed for figure 3.

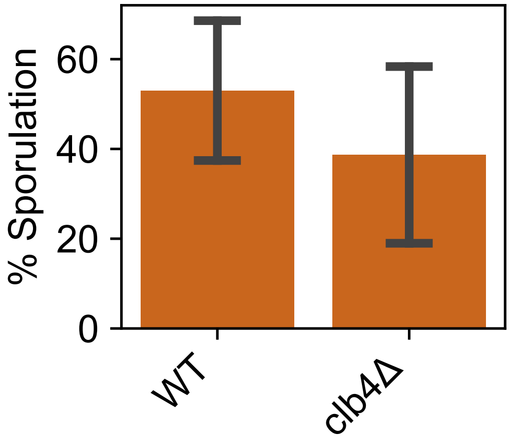

Figure S3. Sporulation fraction of meiotic time course cultures The fraction of sporulated cells (asci) in the cultures assayed for figure 5.

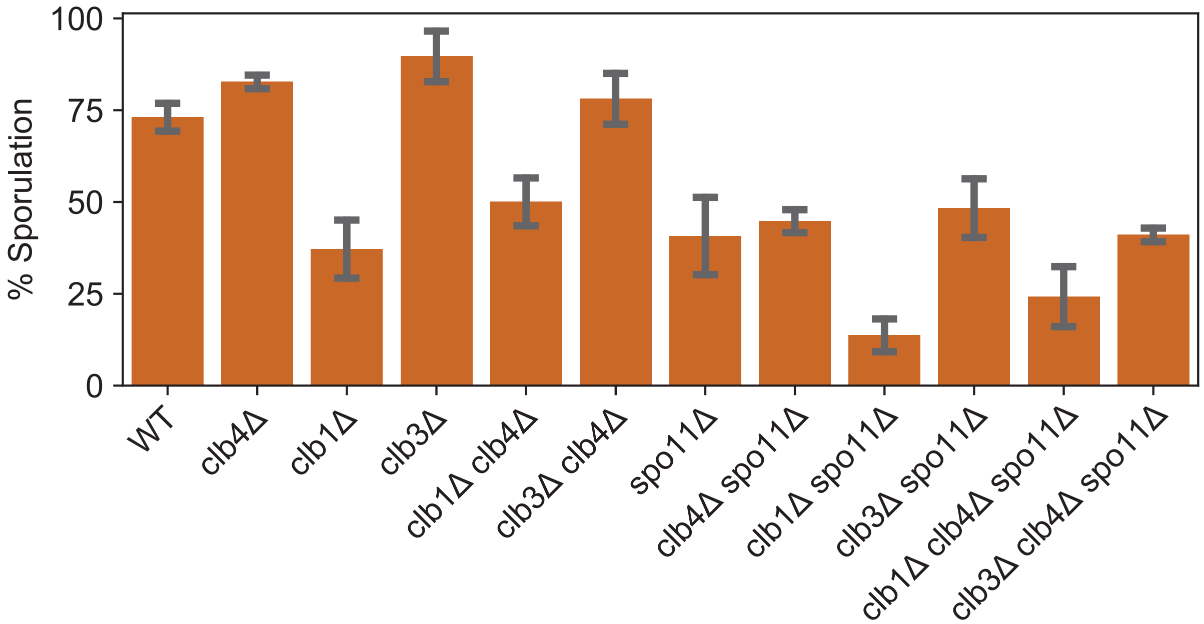

Figure S4. Sporulation fraction of *CLB* mutants. The fraction of sporulated cells (asci) in the cultures assayed for figure 7.

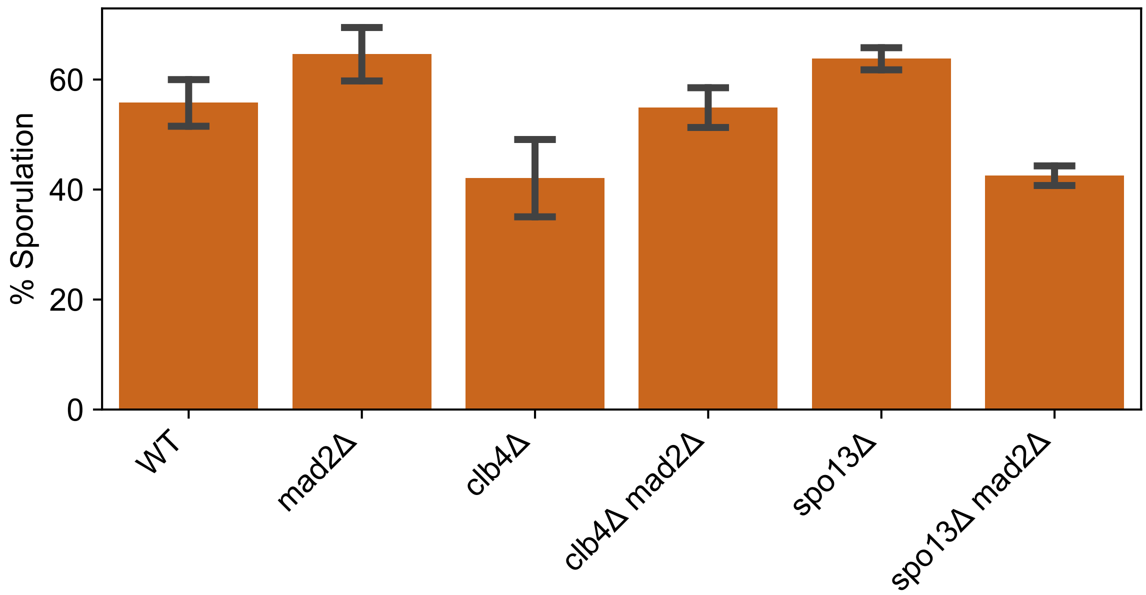

Figure S5. Sporulation fraction of *MAD2* mutants. The fraction of sporulated cells (asci) in the cultures assayed for figure 6.
